# Studying the various facets of emotional aging

**DOI:** 10.3389/fpsyg.2014.01007

**Published:** 2014-09-09

**Authors:** Natalie C. Ebner, Håkan Fischer

**Affiliations:** ^1^Social-Cognitive and Affective Development Lab, Department of Psychology, University of FloridaGainesville, FL, USA; ^2^Department of Psychology, Stockholm UniversityStockholm, Sweden

**Keywords:** emotional aging, emotion perception, emotion-related cognition, emotion experience, emotion regulation, multi-faceted

To study emotional aging is to study a very multi-faceted concept. In particular, the study of emotion and aging covers a wide range of topics. Taking a closer look, domains of functioning can be differentiated such as pertaining to the experiential nature of emotion or its regulation, as well as social-cognitive processes associated with the perception of emotion in others or emotion-related attention and memory retrieval. Importantly, evidence over the last two decades suggests that not all of these functional domains are negatively affected by the aging process. Rather late-life development in emotion-related functional domains is characterized by multi-directionality, in that aging seems to be associated with deterioration in abilities related to emotion perception and increased difficulty remembering (particularly negative compared to positive) emotional information, while emotional experience and emotion-regulatory capacities appear to remain relatively preserved or even improve with age.

Also, the study of emotion and aging covers a wide range of theoretical orientations, paradigms, and methods to adequately capture the complexity of the phenomenon. In particular, emotional aging researchers use self-report, experience sampling, cognitive-behavioral observation, eye tracking, electro- and psychophysiological as well as different functional brain imaging techniques among their assessment tools to ensure recording from the multiple channels emotions can be expressed in, and to shed light on the phenomenon of interest from different angles.

In the current Frontiers research topic *Emotion and Aging: Evidence from Brain and Behavior*, 13 groups of researchers contributed with their expertise, and the included papers reflect the theoretical, methodological, and statistical diversity of this research field. In the following, we briefly introduce each paper contribution. For a more thorough discussion of the articles' theoretical innovation, methodological approach, and scientific advancement contextualized in the broader literature on emotional aging as well as for a constructive reflection about topics untouched in this research topic and future directions the field could take, see Ebner and Fischer (2014) in this Frontiers research topic.

## Emotional experiences

English and Carstensen (2014) used experience sampling in a community sample of young and older adults and underscore the importance of examining emotional aging in everyday life, thereby carefully considering the moderating roles of specific emotions sampled and assessment time during the day.

## Emotion regulation

Allard and Kensinger (2014) compared young and older adults in their use of emotion-regulatory strategies and provide neuroimaging evidence of less efficient cognitive control processing in aging. Also using functional magnetic resonance imaging, Dolcos et al. (2014) showed that older compared to young adults' spontaneous engagement of emotion control regions resulted in emotion-regulatory benefits. Opitz et al. (2014) used a multiple-channel approach that integrated self-reported emotional intensity, expressive behavior, and autonomic physiology and showed that fluid (but not crystallized) cognitive abilities predicted emotion-regulatory success, independent of age.

## Emotion perception

Völkle et al. (2014) took a closer look at the interplay between mood fluctations and perceptions of emotion in others in an adult lifespan sample comprising young, middle-aged, and older adults and report supporting evidence for a mood-congruency effect that was particularly pronounced in older adults. Riediger et al. (2014) demonstrated that young adults outperformed older adults in their ability to identify emotional experiences accompanying smiles. Their findings furthermore show that age effects varied as a function of both the genuineness of smiles and the age of the smiling person. Studying emotion perception in a clinical-dyadic context with Parkinson's Disease patients and neurologically intact age-matched controls, Petrican et al. (2014) showed that the relationship between partner's proficiency in identifying emotions in others and spousal well-being was moderated by neurological status.

## Emotion-related attention and memory

Svärd et al. (2014) examined emotion-related cognition. They confirmed a direct link between subjective ratings of emotional faces and attention to and memory for such faces, independent of age. Across two studies, effects of emotional content of information on working memory was examined. Truong and Yang (2014) observed that emotional targets facilitated working memory, while emotional distracters disrupted performance, for both young and older adults. Pehlivanoglu et al. (2014) confirmed an age-related deficit in unbinding task-irrelevant facial emotion information. Shedding light on the neural correlates of source memory for socioemotional information, Cassidy et al. (2014) reported an age-related shift from dorsal to ventral medial prefrontal cortex activity during encoding that possibly reflects an increased focus on emotionally relevant information in aging.

Across the empirical contributions in this Frontiers research topic a variety of theoretical approaches are discussed. For example, some contributions conceptualize emotions as a multi-dimensional construct (Völkle et al., 2014), while others propose broad differentiation between positive and negative emotions (Pehlivanoglu et al., 2014; Petrican et al., 2014; Truong and Yang, 2014) and/or highlight the impact of other emotion dimensions than valence such as arousal or potency (Dolcos et al., 2014; English and Carstensen, 2014; Pehlivanoglu et al., 2014; Svärd et al., 2014; Truong and Yang, 2014). This research topic also specifically features two novel theoretical perspectives, with potential to inform future research. In particular, Kunzmann et al. (2014) propose a discrete emotions approach that considers multi-directional age differences in the specific emotions of anger and sadness. Synthesizing the literature, Fölster et al. (2014) emphasize the role of considering age-of-face effects in aging research on emotion perception. They conclude that age-congruency effects are particularly crucial in the context of face memory but may only play a minor role in facial emotion perception.

Taken together, this Frontiers research topic showcases the breadth of approaches when studying emotional aging, highlights common themes as well as topical diversity, and proposes avenues for future research. We regard the presented research as clearly underlining the scientific benefits arising from the broad and interdisciplinary perspective that characterizes emotional aging research today. We believe that the theoretical work, innovative empirical paradigms and methodological and statistical approaches presented in this research topic will constitute valuable tools to guide future research toward validation and extension of current research findings. In this spirit, we would like to conclude this introduction by reflecting on a selection of topics that remained untouched in the current research topic, offering great opportunities for exciting future research moving forward in this domain of inquiry. We argue that in order to obtain a comprehensive picture of emotion and aging, brain-behavior processes need to be directly linked to hormonal and genetic as well as contextual and motivational influences, an integration that is currently largely neglected in the literature. Also, a translation of research findings obtained in healthy aging populations to clinical contexts appears crucial to advance research in pathological aging associated with experience, regulation, and processing of emotion (e.g., in depression or apathy). Moreover, to overcome methodological heterogeneity between studies that complicates direct comparison across studies, future research that applies multiple methods to the same sample is warranted. We look forward to seeing this Frontiers research topic inspire exciting new research on emotion and aging in a multi-faceted and integrated manner.

### Conflict of interest statement

The authors declare that the research was conducted in the absence of any commercial or financial relationships that could be construed as a potential conflict of interest.
